# Is There Gender Disparity in RANZCR Radiation Oncology Grants and Prizes Success?

**DOI:** 10.1111/1754-9485.13836

**Published:** 2025-01-21

**Authors:** Daniel Roos, Lisa Milner

**Affiliations:** ^1^ Department of Radiation Oncology, Royal Adelaide Hospital, Adelaide and School of Medicine Adelaide University Adelaide South Australia Australia; ^2^ Royal Australian and New Zealand College of Radiologists Member Engagement and Services Unit Sydney New South Wales Australia

**Keywords:** gender disparity, grants and prizes, radiation oncology, RANZCR

## Abstract

**Introduction:**

Recent RANZCR studies have demonstrated gender disparity in research publication output of both radiation oncology (RO) trainees and specialists, favouring men. The purpose of this project was to examine success rates by gender of grant and prize (G&P) submissions to the RO Research Committee (RORC) to determine if anything needs to be done about the appraisal process to potentially address that disparity.

**Methods:**

College records between 2011 and 2024 (where applicable) were searched by gender for one RO trainee, and two other research manuscript prizes, and two research grant rounds. During that period, the averaged gender ratio for the RO Faculty specialist membership was M:F = 61%:39%. Fisher's exact test *p* < 0.05 was considered significant with respect to gender disparity.

**Results:**

Relative to the gender ratio of applicants, there were no statistically significant gender differences between winners for any of the five G&Ps individually (*p* ≥ 0.15 for each), or in composite (*p* = 0.25). Although application rates overall (M:F = 62%:38%) were consistent with the membership gender ratio, women were markedly less likely to apply for prizes (25% of applicants) than grants (44%).

**Conclusion:**

No gender disparity was found for winners of the five RO G&Ps individually or overall relative to applicant gender ratios. Accordingly, it does not appear that the RORC needs to change its assessment processes in relation to gender. However, women were under‐represented in prize applications, reflecting previously reported gender differences in award‐seeking behaviour.

## 1 Introduction

The Royal Australian and New Zealand College of Radiologists (RANZCR, the College) embraces diversity, equity and inclusion in all its activities [1], including gender equality in research. However, two recent studies have identified significant gender disparities in radiation oncology (RO) research output from both trainees and specialists. Women produce fewer publications than men in any‐authorship and first‐authorship positions per individual during training (*p* = 0.002 and *p* = 0.04, respectively) [2], and fewer any‐ and first‐authorships per individual once qualified (*p* = 0.009 and *p* = 0.04, respectively) [3]. Unfortunately, these findings reflect gender disparity generally in the workplace, in most aspects of medical practice, and in RO in particular, the causes of which are multifactorial including workplace discrimination, sexual harassment, child‐rearing/family responsibilities, and inequitable mentoring, sponsorship and allocation of teaching and clinical work [4, 5, 6].

The RANZCR RO Faculty awards various competitive annual grants and prizes (G&Ps), applications for five of which are appraised by the RO Research Committee (RORC), to encourage excellence in research which is a major priority of the College [7]. The total value of those five G&Ps is typically at least AUD$100 k annually, comprising three RO research publication prizes (in Quality Improvement, Indigenous Health and trainee research, the Bourne & Langlands Prize, each to the value of $2 k), and two research grant rounds (a single Withers & Peters Grant of $25 k, and multiple RO Research Grants of $5 k‐25 k). The Bourne & Langlands Prize is restricted to research undertaken during training but the other G&Ps are currently open to trainees, Fellows and Educational Affiliates (overseas trained ROs registered to practice in RANZCR jurisdictions but not RANZCR Fellows) with the caveats that pre‐Phase 2 examination trainees, Educational Affiliates and RANZCR Fellows > 5 years post‐Fellowship are ineligible for the Withers & Peters Grant and all specialists > 10 years post‐Fellowship are ineligible for the RO Research Grants.

In the light.... Better as: "In light of…" or "In view of…" of the abovementioned gender disparities in publication output, the obvious question is whether anything can (or needs to) be done at the level of assessment of G&P applications to possibly address that inequality. As the identity, and hence gender, of applicants is known to the assessing RORC members, there could be unconscious bias against women (assuming, quite reasonably, that the scientific quality of the applications is, on average, equivalent). Specifically, should the RORC consider allocating bonus scoring points to women applicants to increase their chances of success? This is already done for junior and Indigenous applicants and for projects focusing on disadvantaged groups where one or two extra points may well secure a win for projects otherwise comparable scientifically. Clearly, this would be inappropriate if the award rates of men and women are proportionate to their application rates and gender ratios in the College membership (implying that the observed publication output disparity is due to other causes).

The purpose of this work was to answer that question by examining success rates of men and women RO members in winning prizes and obtaining research funding between 2011 and 2024 (where applicable), a period contemporary enough to be potentially relevant to the publication output disparities and over which (mostly) accurate records are available.

## 2 Methods

This project was approved by the Dean of the Faculty of RO in September 2024. Because no patient data were involved and no individuals are identifiable in the report, Ethics Committee approval was deemed unnecessary.

College records were interrogated by gender for all applications for the five G&Ps between 2011 and 2024. In cases where multiple applications for the same grant or prize were received from the same individual, the corresponding gender was assigned multiple times, so the analysis reflects both the breadth of research productivity across each gender and also the depth (productivity) per individual.

College Annual reports do not provide separate gender data for the RO and Clinical Radiology Faculties, but College staff were able to provide yearly figures from the RO member database over 2019–2024. Because reliable older records were no longer accessible via this means, gender ratios were also obtained from a 2010 College‐wide RO workforce survey [8], from another publication for 2014 and 2018 [6], and calculated from separate Australian and New Zealand figures covering 2017–23 tabulated in Reference [1] (Table 1). It will be seen that, while there has been a gradual decline in the majority of men RO specialists since 2010, the trainee gender ratio has fluctuated. Acknowledging some overlap in these five paired datasets, for the purposes of this study, these percentages were averaged to enable a gender comparison over the whole study period. Those averages were found to be M:F = 52%:48% for trainees and M:F = 61%:39% for specialists (the medians were almost identical to the means) (Table 1).

**TABLE 1:** Data used to obtain average radiation oncology trainee and specialist gender ratios during the study period (% men shown for clarity).

Source	Trainees (% men)	Specialists (% men)
Leung and Vukolova [8] (for 2010)	47	71
Hesselberg et al. [6] (for 2014)	56	61
Hesselberg et al. [6] (for 2018)	49	60
RANZCR [1], averaged over 2017–23	54	57^a^
RANZCR member database, averaged over 2019–24 (range)	52 (51–53)	57^a^ (56‐59^a^)
Overall mean (median)	52 (52)	61 (60)

^a^ Active radiation oncologists, working full or part time.

The RORC had four men Chairs during the 14 years of the study, but the overall gender ratio varied considerably. For example, with an average of 11 members over the last 8 years, the representation of women ranged between 17% and 50% (average 35%, median 36%) [1]. Hence, the committee typically has more men than women together with a man as Chair.

The primary endpoints for this study were the success rates of women and men relative to their application rates for each G&P and for all five G&Ps overall, compared using Fisher's exact test. *p* < 0.05 was considered statistically significant. Non‐significance would constitute indirect evidence that any potential bias in the assessment process was unlikely to be contributing substantively to the observed publication gender disparity, and consequently, modification of the current RORC scoring system would not be justified. Also of interest were application rates by gender overall, and for combined G&Ps, relative to the membership.

## 3 Results

Data summarising applications and success for the five G&Ps by gender are shown in Table 2. Note that all applicants for each G&P self‐identified as either man or woman.

**TABLE 2:** Summary data for applicants and winners of the five radiation oncology annual grants and prizes over 2011–2024 (where applicable).

Grants and prizes	Applicants		Winners		*p* ^a^
	Men	Women	Men	Women	
Bourne & Langlands Prize	34	15	6	6	0.15
Quality Improvement Project Prize	16	1	3	1	0.24
Indigenous Health Research Prize	1	1	1	1	1.0
All three Prizes combined	51	17	10	8	0.053
Withers & Peters Grant	24	18	11	4	0.19
Radiation Oncology Research Grants	55	45	31	27	0.84
Both Grants combined	79	63	42	31	0.74
Overall	130	80	52	39	0.25

^a^ Fisher's exact test.

### 3.1 Prizes

Formal application by interested trainees for the Bourne & Langlands Prize were required from 2013 with 49 applications since, 34 (69%) from men and 15 (31%) from women. Of the 12 winners, 6 of 34 men (18%) were successful compared with 6 of 15 women (40%) (*p* = 0.15).

The Quality Improvement Project Prize was instituted in 2020 with 17 applications since, 16 (94%) from men and 1 (6%) from a woman. No prize was awarded in 2022. Three of four winners in the other years were men. This difference was not statistically significant taking into account the applicant gender ratio (*p* = 0.24).

The Indigenous Health Research Prize was also instituted in 2020 with two successful applications since (2020 and 2023), one of each gender (*p* = 1.0), both non‐Indigenous.

### 3.2 Grants

Two individuals shared the Withers & Peters Grant in 2011, two also in 2012 but no prize was awarded in 2023. There was one winner in each of the remaining years. Of the 42 applications between 2011 and 2024, 24 (57%) were from men and 18 (43%) from women. Of the 15 winners, 11 of 24 men (46%) were successful compared with 4 of 18 women (22%) (*p* = 0.19).

Between 2011 and 2024, there were 100 applications for RO Research Grants, 55 from men and 45 from women. Of the 58 winners, 31 of 55 men (56%) were successful compared with 27 of 45 women (60%) (*p* = 0.84).

### 3.3 Grants and Prizes Overall

The applicant gender ratio for the five G&Ps overall was 130:80 = 62%:38% (noted to be very close to the averaged specialist RO membership ratio of 61%:39%). Of the 91 winners, 52 of 130 men (40%) were successful compared with 39 of 80 women (49%) (*p* = 0.25), clearly *not* favouring men. This was also the case for the Bourne and Langlands Prize, the only one of the five G&Ps restricted to trainee applicants (see above).

The M:F applicant ratios for all three prizes combined versus both grants combined were 51:17 (75%:25%) vs. 79:63 (56%:44%), respectively. Of the 18 prize winners, 10 of 51 men (20%) were successful compared with 8 of 17 women (47%) (*p* = 0.053). Of the 73 grant winners, 42 of 79 men (53%) were successful compared with 31 of 63 women (49%) (*p* = 0.74).

## 4 Discussion

We found no evidence of gender disparity in success rates for the five RORC assessed G&Ps (*p* ≥ 0.15 for all five of them individually and overall). This is particularly noteworthy, given the predominance of men on the RORC (typically about two thirds of committee members) and solely men Chairs over the study period. The implication is that the previously observed differences in publication output of both trainees and specialists by gender must be due to other factors, and there needs to be no adjustment to the current RORC scoring system. Indeed, it could be argued that applying bonus points for women applicants would inappropriately disadvantage men.

Whilst G&P application rates overall (62%:38%) were consistent with the RO Faculty specialist gender ratio (61%:39%), there were notable differences between the three prizes versus the two grants combined—women were markedly under‐represented in prize applications (25%), whereas those for grants were actually slightly in favour of women relative to the membership (M:F = 56%:44%). It is well known that women are less likely to self‐promote and often harbour the ‘imposter syndrome’ [6]. Hence, while women members are applying for grants to try and engage in research, they may be less likely to perceive themselves as deserving of accolades, be encouraged to nominate by peers, or to justify the time needed to apply for such awards (perhaps a combination of each of these factors). Interestingly, those women who did apply for prizes were quite successful (8 of 17, 47%), almost statistically significantly so relative to men (10 of 51, 20%) (*p* = 0.053), while success rates for Grants were very similar (M:F = 53%:49%, *p* = 0.74). Since the numbers of prize applicants and winners were low (two of the three prizes only commenced in 2020), more time will be needed to determine whether this apparent difference is real.

A RANZCR study in 2023 reported that the gender distribution of RO “Fellows” receiving a composite total of 254 grants, awards and prizes (including others not scored by the RORC, and so not analysed here) between 2010 and 2020 was M:F = 56%:44% [6]. As shown in Table 1, for 2 years within that period (2014 and 2018) the proportion of men Fellows was stable at 60%–61%. Hence, there was no signal of G&P success in favour of men, entirely consistent with our findings. However, there was no explicit comparison relative to the gender ratio of applicants, no breakdown of outcomes for individual G&Ps and (presumably) exclusion of trainee G&Ps in the earlier study.

Corresponding data from overseas RO organisations are limited. A 2022 US study on National Institutes of Health (NIH) funding for RO between 2016 and 2019 found that 75% of recipient Principal Investigators were men [9]. This was not compared to the gender ratio of actual or potential applicants, but it did echo earlier work on NIH funding at 82 ‘academic RO departments’ where the overall ratio of faculty members receiving grants was M:F = 19%:11% (*p* < 0.05) [5]. Another study in 2024 reported that between 2010 and 2022, the proportion of women faculty members in nominating programs who were recipients of the American Society for Radiation Oncology (ASTRO) Association of Residents in RO ‘Educator of the Year Award’ averaged 25%, under‐representing the proportion of women faculty in 11 of the 13 years, although there *was* a statistically significant increase in women recipients during the study period (*p* < 0.01) [10]. At a higher award level, between 2005 and 2017, women received only 2 (6%) of 32 ASTRO Gold Medals [11]. A single centre report (Massachusetts General Hospital) on philanthropic support in academic oncology over 10 years for both medical oncologists (80% of the sample) and ROs (20%), found that men overall were significantly more likely to receive donations (70% vs. 30%, *p* = 0.034), had a higher total number of donations (median 43 vs. 1, *p*‐value not stated) and obtained significantly higher median amounts ($260 k vs. $37 k, *p* = 0.019) [12]. Although the RO data were not reported separately, on multivariable analysis overall, academic rank and *h*‐index were significantly associated with philanthropic funding, but not gender per se. This implies that inequities in promotion and productivity are likely responsible for the observed differences, mirroring findings previously reported for other aspects of RO gender disparity [4, 5, 9]. Indeed, this may well also account for the abovementioned RANZCR RO publication output findings too, although presumably cultural differences contribute to the apparent international discrepancy in G&Ps success by gender.

There are several limitations to this study. Firstly, there is some uncertainty in the applicability of reported RO specialist numbers and gender ratios because of varying inclusion criteria (Fellows, active Fellows, retired Fellows, overseas Fellows, Honorary Fellows, Life Members, Educational Affiliates) which are not always specified in source documents. Further, eligibility for the individual G&Ps differs substantially, and does change over time, both of which may impact upon appropriate denominators for each. The averaging of gender ratios over five datasets here (Table 1) was intended to provide pragmatic approximations for trainee and specialist gender ratios over the 14‐year study period to enable comparison of expected with observed success rates. Secondly, we acknowledge that other variables, such as jurisdiction and minoritized ethnicity, could influence application and success rates but were not the focus of this work. Thirdly, we did not examine outcomes for several other RO awards, prizes and fellowships outside the remit of the RORC—these, too, *might* be subject to gender disparity due to non‐blinding of applicant identities. Fourthly, both the RANZCR Clinical Radiology and RO Faculties function under the same gender equity principles, but we did not attempt to make any inter‐Faculty G&P comparisons. Finally, in common with other gender disparity research, we applied self‐reported dichotomised gender, but do acknowledge the reality that it exists instead on a spectrum.

In conclusion, this examination of success in five RANZCR RO G&Ps found no evidence of gender disparity, so it appears that college efforts to address the previously observed publication output gender differences need to be directed elsewhere than the G&P assessment process.

## 5 Ethics Statement

Ethics Committee approval was waived.

## Consent

The authors have nothing to report.

## Conflicts of Interest

Daniel Roos is Chair of the RANZCR Radiation Oncology Research Committee (RORC) at the time of writing. Lisa Milner is a RANZCR Project Officer and RORC co‐ordinator.

## Data Availability

The data that support the findings of this study are available from the corresponding author upon reasonable request.
